# Long-term microfluidic tracking of coccoid cyanobacterial cells reveals robust control of division timing

**DOI:** 10.1186/s12915-016-0344-4

**Published:** 2017-02-14

**Authors:** Feiqiao Brian Yu, Lisa Willis, Rosanna Man Wah Chau, Alessandro Zambon, Mark Horowitz, Devaki Bhaya, Kerwyn Casey Huang, Stephen R. Quake

**Affiliations:** 1 Department of Electrical Engineering, Stanford University, Stanford, CA 94305 USA; 2 Department of Bioengineering, Stanford University, Stanford, CA 94305 USA; 3 Sainsbury Laboratory, Cambridge University, Cambridge, CB2 1LR UK; 4 Department of Industrial Engineering, University of Padova, Padova, 35131 Italy; 5 Department of Plant Biology, Carnegie Institution for Science, Stanford, CA 94305 USA; 6 Department of Microbiology and Immunology, Stanford University School of Medicine, Stanford, CA 94305 USA; 7 Chan Zuckerberg Biohub, San Francisco, CA 94158 USA

**Keywords:** Cyanobacteria, Microfluidics, Single-cell imaging, Light-dark cycles, Cell-size homeostasis, Circadian clock, Photosynthesis

## Abstract

**Background:**

Cyanobacteria are important agents in global carbon and nitrogen cycling and hold great promise for biotechnological applications. Model organisms such as *Synechocystis sp*. and *Synechococcus sp*. have advanced our understanding of photosynthetic capacity and circadian behavior, mostly using population-level measurements in which the behavior of individuals cannot be monitored. *Synechocystis sp*. cells are small and divide slowly, requiring long-term experiments to track single cells. Thus, the cumulative effects of drift over long periods can cause difficulties in monitoring and quantifying cell growth and division dynamics.

**Results:**

To overcome this challenge, we enhanced a microfluidic cell-culture device and developed an image analysis pipeline for robust lineage reconstruction. This allowed simultaneous tracking of many cells over multiple generations, and revealed that cells expand exponentially throughout their cell cycle. Generation times were highly correlated for sister cells, but not between mother and daughter cells. Relationships between birth size, division size, and generation time indicated that cell-size control was inconsistent with the “sizer” rule, where division timing is based on cell size, or the “timer” rule, where division occurs after a fixed time interval. Instead, single cell growth statistics were most consistent with the “adder” rule, in which division occurs after a constant increment in cell volume. Cells exposed to light-dark cycles exhibited growth and division only during the light period; dark phases pause but do not disrupt cell-cycle control.

**Conclusions:**

Our analyses revealed that the “adder” model can explain both the growth-related statistics of single *Synechocystis* cells and the correlation between sister cell generation times. We also observed rapid phenotypic response to light-dark transitions at the single cell level, highlighting the critical role of light in cyanobacterial cell-cycle control. Our findings suggest that by monitoring the growth kinetics of individual cells we can build testable models of circadian control of the cell cycle in cyanobacteria.

**Electronic supplementary material:**

The online version of this article (doi:10.1186/s12915-016-0344-4) contains supplementary material, which is available to authorized users.

## Background

Cyanobacteria are ancient oxygenic photoautotrophs with important roles in global carbon and nitrogen cycles, and hold promise as chassis organisms for products such as biofuels [1]. Cyanobacteria possess a circadian clock and cell-cycle regulation that allow them to robustly respond to diel cycles. Synchronized populations of the unicellular cyanobacterium *Synechococcus elongatus* PCC7942 have been used to identify the main components responsible for circadian oscillations [2]. Another model species, *Synechocystis* sp. PCC6803 (hereafter *Synechocystis*), has played an important role in elucidating photosynthetic pathways [3] and phototaxis [4–6], in addition to providing insight into circadian cycle regulation [7, 8]. *Synechocystis* can be engineered to produce many biomolecules [9]. However, it remains unknown how the cell cycle is coupled with growth (here referring to volume expansion) in single cells and across generations and how this coupling is influenced by diel cycles. A detailed understanding of the phenotypic heterogeneity across populations and how environmental factors such as rapid changes in light affect growth may provide insight into how cells integrate external stimuli with internal mechanisms of cell-cycle and cell-size regulation. This understanding will also be required for optimizing the efficiency of large-scale *Synechocystis* bioreactors.

Bacteria typically maintain a size and shape that is characteristic of the species, suggesting that cell-size control is fundamental across the kingdom. Most studies of bacterial growth have focused on fast-growing heterotrophs such as *Escherichia coli* [10], *Caulobacter crescentus* [11], *Bacillus subtilis* [12], and *Pseudomonas aeruginosa* [13], which differ in many respects from slow-growing cells such as *Synechocystis*. Recently, microscopy has been used to track single fast-growing cells on agar pads or in microfluidic devices and to characterize correlations between cell size and generation time (defined as the time between cell birth and cell division). For several organisms, studies have demonstrated that size homeostasis is maintained via an adder rule whereby cells increase by a constant volume each generation regardless of birth size [11]. These studies have focused almost entirely on rod-shaped bacteria with short generation times of less than 1 h; it remains to be seen whether similar homeostatic behaviors are exhibited by cells with other morphologies and/or much longer doubling times.

Several technical challenges complicate the single-cell microscopy-based analysis of slow-growing cocci such as *Synechocystis*. Although their small size (1–2 μm) is typical of many model bacteria, *Synechocystis* and other cyanobacteria require light and carbon dioxide for photosynthesis. Evaporation makes hydrogel surfaces unfit for long-term tracking of slow-growing cells. Microfluidics alleviates problems associated with evaporation, but devices can be difficult to use, particularly in high throughput, due to lack of automation and system-level integration of a comprehensively controlled microfluidic system including microscope, stage, image acquisition, and actuation of microfluidic valves. In addition, some microfluidic devices have been designed to exploit the elongation of rod-shaped cells along only one direction [14, 15]; such one-dimensional expansion is unlikely to be the case for many non-rod-shaped organisms and hence mechanical constraint within a micron-sized channel would not reflect normal growth. To address these issues, we modified a microfluidic cell-culture system for monitoring *Synechocystis* growth and division over several generations in continuous illumination or with light-dark cycling [16]. We determined that cells undergo exponential growth during times of illumination, with expansion and division almost completely inhibited in the dark. Sister-cell pairs exhibited highly correlated generation times, even maintaining synchrony throughout dark periods. By comparing our experimental data to simulations of various cell-size control models, we found that *Synechocystis* cells are unlikely to follow the ‘sizer’ or ‘timer’ models; instead, the ‘adder’ rule of constant volume increment better explains the observed trends. In summary, our analyses reveal how light plays a critical role and is tightly integrated with the *Synechocystis* cell cycle.

## Results

### Microfluidics and probabilistic image analysis facilitate long-term quantification of growth behavior

**Fig. 1:**
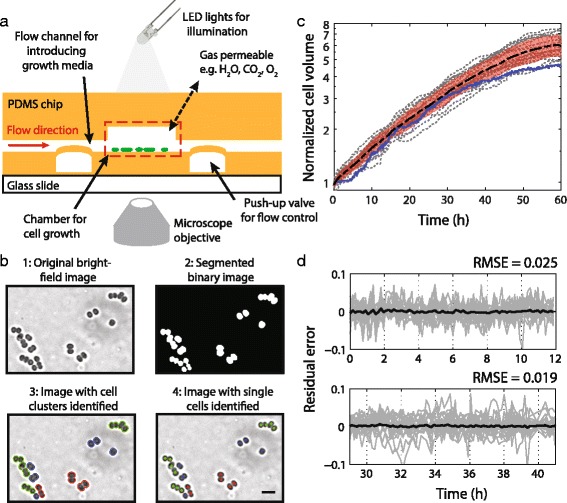
Microfluidic bacterial culture setup and analysis empowers long-term analysis of *Synechocystis* growth and division. **a** Cross-section of the microfluidic cell culture chip. Top flow layer contains cyanobacterial cells. Flow can be controlled using push-up valves. Setup was modified to enable automated control of LED illumination. Gases, including CO_2_, can diffuse into the cell culture chambers. **b** Imaging analysis pipeline, in which the original image (1) is first segmented into a binary image (2), from which cell clusters are identified (3), and then further segmented into single cells whenever possible (4). For each single cell identified in a cluster, the contour defining the interior and the location of the center are determined. Scale bar: 5 μm. **c** Each gray line represents the growth trajectory of one *Synechocystis* lineage starting from a single cell, normalized to the initial cell volume. The mean normalized growth (*black*) and standard deviation (*shaded orange*) are shown. Total cell number (*blue*) based on the automated image analysis pipeline in (**b**) increases at the same rate as total lineage volume for the first 40 h. **d** Residuals from exponential fits of individual lineage growth curves (*gray*) during the first 12 h of growth (*top*) and between 29 and 41 h (*bottom*) exhibited small root mean square error (RMSE), demonstrating exponential growth. The mean of all residuals is shown in black

To determine how the growth and division of *Synechocystis* cells vary over time and across light/dark cycling regimes, we augmented an existing microfluidic cell-culture system [16] with a switchable light input (Fig. 1a, Additional file 1: Figure S1). Our system has 96 chambers, allowing for multiple observations to be carried out in parallel. Furthermore, the system has several features that are beneficial for culturing and imaging bacteria: (1) cells are not required to grow in one dimension or divide along the same axis; (2) phototrophs that require light as an input in addition to nutrients can be studied; (3) slow-growing species can be maintained without evaporation or loss of focus for extended periods; and (4) experimental throughput can be dramatically enhanced by imposing different growth conditions on the same device.

The coccoid shape and small size of *Synechocystis* cells make robust identification of cell division events challenging. To address this, we developed an automated image analysis pipeline to track cell positions and to identify newly divided sister cells in a set of time-lapse frames (Fig. 1b, Additional file 2: Figure S2). The key advantage of our analysis method is a probabilistic framework specifically trained on *Synechocystis* morphologies (Additional file 3: Figure S3, Additional file 4). This framework avoids hard thresholds that define cell boundaries and division events, and allows for correction of classification errors using information from the changes in cell shape over time. Moreover, in cases where a pair of cells is not accurately segmented, the algorithm still classifies the cluster as distinct from a single cell, avoiding lineages with artefactually high division times due to missing the division event. Our image analysis method can operate solely on bright-field or phase-contrast microscopy images, eliminating the dependence on fluorescence images for cell segmentation. This aspect is particularly important for cyanobacteria, which exhibit high levels of auto-fluorescence. In general, removing the requirement of fluorescence imaging also avoids potential inhibition of cell growth due to fluorescence excitation [17], or frees up the fluorescence channel for other applications.

To determine the growth dynamics of *Synechocystis* cells over multiple generations, we estimated the volume of individual cells by assuming rotational symmetry of the cell contour (Additional file 5: Figure S4) and tracked cell lineages from the single-cell stage for 60 h in 20 different chambers (Fig. 1c, Methods). We observed that all cells grew, though at different rates (Additional file 6: Figure S5A). Total volume of all lineages, normalized to the volume of the initial cell in the first frame, increased approximately exponentially for the first 40 h (Fig. 1c). Mean residuals after fitting two separate sections of the lineage growth curve further confirmed exponential growth (Fig. 1d). At later times, lineage growth rate slowed down, presumably reflecting the consumption of nutrients in the medium as cell density increased over the course of the experiment. To determine whether rates of division were coordinated with lineage growth rates, we automatically counted the number of cells in each lineage over time and found that mean cell number increased at the same rate as the mean lineage volume (Fig. 1c), suggesting cell-size homeostasis. The deviation between mean cell number and mean volume in the final 20 h is due, at least in part, to the presence of clusters with many cells in which accurate number quantification is challenging. Regardless, the combination of our experimental and analysis platforms enables rapid and robust quantification of bacterial growth and division across multiple days, empowering long-term single-cell analyses of slow-growing species and ellipsoidal cells such as *Synechocystis*.

### *Synechocystis* cell volume expands exponentially under continuous light

**Fig. 2:**
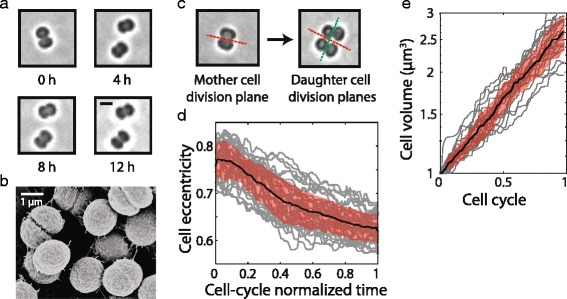
*Synechocystis* cells expand exponentially under continuous illumination. **a** Representative time-lapse images showing growth and division of a pair of *Synechocystis* sister cells. Scale bar: 2 μm. **b** Scanning electron micrograph showing ellipsoidal *Synechocystis* cells, with some undergoing divisions, all of which are approximately symmetric. **c** Division planes in daughter cells are always perpendicular to the division plane of the mother cell. **d** Cell eccentricity (ratio of minor axis length to major axis length) as a function of normalized time during the cell cycle. Each gray line represents one cell, with the mean (*black*) and one standard deviation around the mean (*orange shaded area*) overlaid. **e** Single-cell growth (volume expansion) curves (*gray lines*) normalized to the generation time and plotted on a log scale with mean (*black*) and standard deviation (*shaded orange area*) overlaid



We examined the dynamics of *Synechocystis* cell shape and volume over the cell cycle. Cells expanded in volume throughout the cell cycle, and constriction was evident early in the cell cycle for most cells (Fig. 2a, b). Cell divisions were approximately symmetric in most cases; the standard deviation of sister cell size mismatch at birth was 3.3%. Daughter cell division planes were always perpendicular to the mother cell division plane (140/140 cells, Fig. 2c), as previously reported [18] and similar to other cocci such as *Staphylococcus aureus* [19] and *Neisseria gonorrhoeae* [20]. Although traditionally thought of as spherical, *Synechocystis* cells were ellipsoidal and exhibited a characteristic eccentricity trajectory during the cell cycle, independent of generation time (Fig. 2b, Additional file 6: Figure S5B, C). At birth, cells had a minor to major axis ratio of 0.77 ± 0.04. This ratio decreased monotonically to 0.63 ± 0.03 at the time of division (Fig. 2d). These two values are approximately consistent since, upon symmetric division, the new daughter cells of a mother cell with ratio 0.63:1 would be predicted to have a ratio of 0.5/0.63 = 0.79, further substantiating our observation that consecutive division planes are perpendicular to one another. After the completion of cytokinesis, some daughter cells moved apart over a time period of 10–20 min, ending up separated by a gap of a few microns (Additional file 7: Movie S1); this separation was more prevalent for isolated doublets than for clusters of four or more cells.

**Additional file 7: Movie S1. **Time-lapse video showing three sets of Synechocystis sister cells, grown under continuous illumination, moving apart by a few microns after division. This movement usually takes place in 10-20 min, which is short compared to the mean generation time (16.9 h), and is more prevalent for isolated doublets than for larger clusters of cells.


The exponential growth of a microcolony (Fig. 1c, d) does not automatically imply exponential growth of individual cells over the cell cycle. To examine whether single cells also expanded their biomass exponentially or underwent distinct growth phases during their cell cycle, we quantified the volume of single cells for which boundaries could be confidently identified throughout their cell cycle (*n* = 140, Additional file 8: Movie S2). Most cells continuously increased in volume exponentially throughout the cell cycle under continuous illumination (Fig. 2e), even though lineage growth eventually slowed during the experiment, suggesting that they were growing in a relatively constant environment throughout their cell cycle. Therefore, our microfluidic device supports exponential expansion of cells and cell populations over multiple days and multiple cell-division events.



**Additional file 8: Movie S2. **Time-lapse video tracking two cell cycles of growth and division under continuous illumination starting from a single Synechocystis cell. Red boundaries outline single cells extracted by our image analysis pipeline.


### Growth and division of *Synechocystis* cells are rapidly inhibited in the dark

Unlike most heterotrophic fast-growing bacterial species whose growth has been characterized at the single-cell level, cyanobacteria divide relatively slowly, rely on photosynthesis for energy, possess a robust circadian cycle, and respond to environmental light stimuli [21]. Thus, it is important to determine the growth dynamics of *Synechocystis* cells under light-dark cycles that are similar to conditions encountered in the environment. Most previous studies have entrained cyanobacteria using light-dark cycles and then observed free-running behavior under continuous illumination [22]; however, this strategy does not reveal how quickly cells respond to changes in light conditions or if there is heterogeneity in cellular responses. Our microfluidic culture system has the advantage of allowing direct observation of *Synechocystis* cells during the dark phase, using short (millisecond) pulses of low-intensity light to record bright-field images (Additional file 4).

**Fig. 3:**
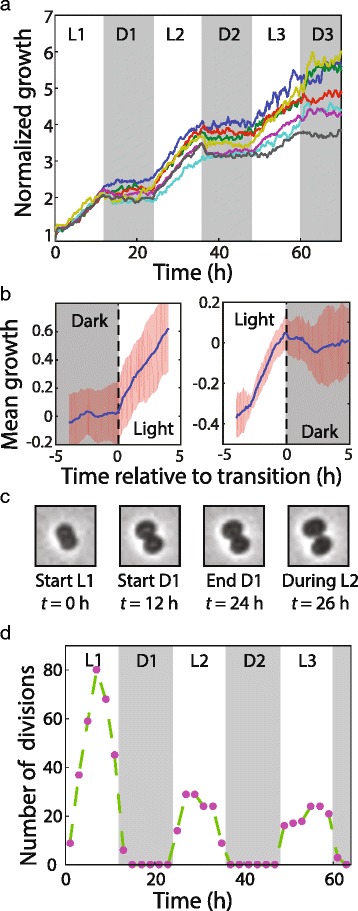
*Synechocystis* expansion and division rapidly pause and restart during light-dark cycles. **a** For lineages under light-dark cycles starting from single cells, the total volume of all cells in the lineage, normalized to the volume of the initial cell, shows that cells expanded only during light periods. L1, L2, and L3 and D1, D2, and D3 represent illuminated and dark periods, respectively. **b** Mean growth curves over all lineages and cycles demonstrate that cells started to expand immediately after entry into light periods (*left*) and rapidly halted expansion after entry into the dark (*right*). Standard deviation is shown in orange. **c** Time-lapse images of a cell in the process of constriction before the transition from light to dark. Constriction halted in the dark and continued when illumination resumed after the dark interval. **d** Number of division events observed during each period of light-dark cycles shows that divisions were not biased toward the beginning or end of light intervals



We cultured *Synechocystis* cells under 12-h light-dark cycles for 3 days and extracted volumes of single cells and lineages from time-lapse images. *Synechocystis* cells grew continuously during the light phase, as we observed in continuous illumination conditions (Fig. 2e), but strikingly, there was minimal volume expansion in the dark (Fig. 3a). More specifically, expansion was restricted specifically to periods of illumination across all microfluidic chambers and ceased completely in all tracked lineages during the dark period (Additional file 9: Figure S6A). During transitions from light to dark or dark to light, cells stopped and restarted growth, respectively, without any detectable delay (within the ~10-min resolution of our imaging) (Fig. 3b). Interestingly, cells dramatically increased their motility during the dark periods (Additional file 10: Movie S3), suggesting that cells still retain enough energy to move despite the absence of growth. The residual errors from exponential fits to lineage growth curves during the first two illumination periods indicated that cells grew exponentially in the light even with the intervening period of growth stoppage in the dark (Additional file 9: Figure S6B). Moreover, the absence of growth while imaging in the dark indicates that the short pulses of light necessary to obtain bright-field images do not induce detectable levels of cellular growth.

**Additional file 10: Movie S3. **Time-lapse video comparing Synechocystis movement during an illumination period (left) with a dark period (right). Left and right panels represent the same area of the same microfluidic chamber. Growth and division are apparent under illumination, while increased movement occurs in the dark period.


In the dark, cell division also halted, even in those cells with substantial constriction prior to the LED being switched off (Fig. 3c, Additional file 11: Movie S4). In the subsequent illumination period, cells completed cytokinesis. Only 6/547 (1%) of division events were observed in the dark, all of which occurred within 30 min after the light was turned off (Fig. 3d). The timing of division events displayed no preference for the beginning or end of the illuminated intervals. There was a peak of division events in the middle of the first interval, while the distribution was approximately uniform in the second and third intervals (Fig. 3d). We observed an increase in the number of divisions in the first light interval that stabilized by the second light interval. The initial increase was largely due to a burst of divisions that occurred once cells began incubation in the device. We do not know the origin of this synchronization, but we note that the first division event for each cell does not contribute to our generation time statistics because we can only measure birth time after the first division has taken place. Taken together, our results indicate that light is necessary for both growth and division of *Synechocystis* cells.



**Additional file 11: Movie S4. **Time-lapse video of a single Synechocystis cell grown under light-dark cycles. Cell growth and division do not occur during dark periods, but resume rapidly after light is restored.


### Sister cells have similar generation times whether grown under continuous light or light-dark cycles

**Fig. 4:**
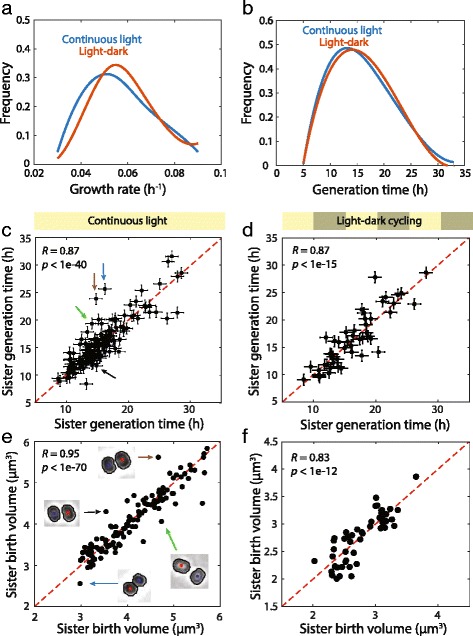
Sister cell generation times are strongly correlated after symmetric divisions. **a**, **b** Distributions of growth rates (**a**) and generation times (**b**) are similar when comparing cells under continuous illumination and light-dark cycles. Generation times are defined as the interval from birth to division. For cells grown under light dark cycles, generation times were calculated based on ignoring the dark periods during which no growth was observed (Fig. 3b). **c**, **d** Generation times of sister cells are highly correlated under continuous illumination (*N* = 139 sister pairs in (**c**)) and light-dark cycles (*N* = 48 in (**d**)). Error bars represent uncertainties in the exact moment of division. **e**, **f** Birth volumes of sister cells are highly correlated under continuous light (*N* = 139 in (**e**)) and light-dark cycles (*N* = 48 in (**f**)). In (**e**), images of sister cells resulting from asymmetric divisions are highlighted by colored arrows, with the corresponding data point in (**c**) indicated by an arrow of the same color and showing large differences in generation times

In addition to examining the instantaneous growth kinetics of lineages and single cells, our data also enabled interrogation of the timing of cell division and the coupling of division to cell size. Generation times (*T*) and volumes at birth (*V*_b_) and division (*V*_d_) were extracted from single-cell growth curves (Additional file 12: Figure S7A), with generation times in our light-dark cycle experiment defined as the time spent in the light since cells did not increase in size or divide during dark periods (Additional file 12: Figure S7B, Methods). Under continuous illumination, there was a wide range of single-cell generation times from 5 to more than 30 h with a mean of 16.9 h, approximately consistent with the mean growth rate 0.055 h^–1^. Surprisingly, the introduction of dark periods had no impact on the distributions of growth rates (Fig. 4a) or generation times (Fig. 4b). Through visual inspection of all time-lapse movies, we confirmed that uncertainties in the timing of division events (~1 h) were not the cause of variation in generation times. In continuous light, there was a highly significant correlation between sister cell generation times (*R* = 0.87, *P* < 1 × 10^–39^, Fig. 4c), suggesting that the observed variation in generation times across all cells was not entirely stochastic. The correlation persisted when the data was split temporally into the first and second halves of the experiment based on when sister division occurred, indicating that the slowdown in growth in the second half of our experiment was not the underlying cause of the correlation (Additional file 13: Figure S8A, B). During light-dark cycles, sister-cell generation times (*R* = 0.87, *P* < 1 × 10^–14^, Fig. 4d) remained highly correlated, indicating that after suspension of growth and division in the dark cells promptly resumed the process that determines generation time. By contrast, mother and daughter generation times were not correlated (*R* = –0.10, *P* = 0.59; Additional file 13: Figure S8C).

To extract single-cell growth related parameters from experiments under light-dark cycles, we ignored intervals of single-cell growth curves in the dark, in which neither growth nor division was observed (Methods). While the distributions of growth rates and generation times were similar under light-dark cycles compared with continuous illumination, the distribution of cell sizes was slightly smaller under light-dark cycles (Additional file 14: Figure S9). Cell birth and division volume distributions had coefficients of variation of 0.12 and 0.13 in continuous light and 0.15 and 0.17 in light-dark cycles, respectively, in close agreement with the coefficients of variation reported for other bacterial species [23]. Sister cell birth volumes were also highly correlated, indicating that cells generally divided symmetrically, in both continuous light (*R* = 0.95, *P* < 1 × 10^–69^, Fig. 3e) and light-dark cycles (*R* = 0.83, *P* < 1 × 10^–11^, Fig. 4f). Nonetheless, there were a few cells that divided asymmetrically (8/139 cell pairs with birth volume asymmetry > 7%) (Fig. 4e). Interestingly, the resulting daughter cell pairs exhibited large differences in division timing (Fig. 4c), indicating that division asymmetry may influence the ability of daughter cells to maintain their otherwise synchronized generation timing. In summary, the striking similarities between sister cell generation times under continuous light and light-dark growth conditions suggest that the underlying regulatory mechanism is suspended in the dark but otherwise unaffected by light input.

### *Synechocystis* cell-cycle statistics are not consistent with regulation of division timing based on fixed division size or cell-cycle interval

**Fig. 5:**
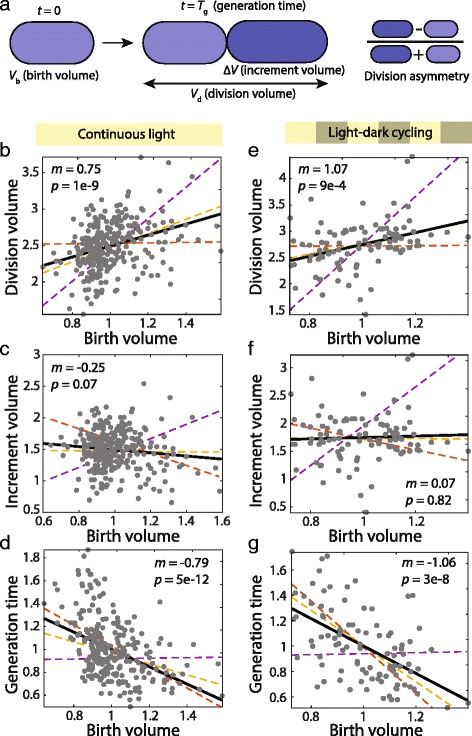
*Synechocystis* expansion and division statistics are most consistent with an adder model for cell-size regulation. Using distributions of birth sizes, division asymmetry, and growth rates extracted from experimental data (*n* = 278 cells for continuous and *n* = 96 for light-dark cycles), simulations of cell growth using the sizer (orange), timer (purple), and adder (yellow) models were performed. Slopes of relationships between growth statistics were extracted from simulations and compared with experimental data (gray circles) and their least square linear fit (black). **a** Schematic illustrating birth volume, volume increment during the cell cycle, division volume, generation time, and division asymmetry. **b**–**g** Relationships between birth volume and division volume (**b**, **e**), volume increment (**c**, **f**), and generation time (**d**, **g**) are most consistent with the adder model. **b**–**d** were determined for cells grown under continuous illumination, and (**e**–**g**) are for cells under light-dark cycles. All volumes were normalized to the mean birth volume. Generation time was normalized to the mean generation time

Like most bacteria, *Synechocystis* cells have a characteristic size that suggests active coupling of growth and division to maintain that size. Various models have been proposed to explain how bacterial cells regulate cell size and generation times via growth and division [24–26]. The three major models are (1) the sizer model, in which cells divide after reaching a fixed size; (2) the timer model, in which cells divide after a fixed time interval; and (3) the adder model, in which cells divide after increasing their volume by a fixed amount. Recent studies have found that several bacterial species [27], as well as budding yeast [28], follow the adder model. To distinguish between these models, we determined the slopes of pairwise relationships between sister-cell birth volume asymmetry (i.e., difference between sister cell quantities normalized by their sum) and cell cycle-related parameters (Fig. 5a) such as generation time and birth, increment, and division volumes.

Under ideal conditions (constant mean cell size and normally distributed growth rates that are independent of cell size), the sizer model predicts that division volume should be independent of birth volume, while the adder and timer models predict slopes of +1 and +2, respectively. However, the expected values of these slopes are altered somewhat due to experimental noise and deviations from ideal conditions. To incorporate how distributions of our measured quantities modify the predicted slopes, we extended a governing set of equations to take into consideration imperfect distributions of various single-cell growth parameters (Additional file 4) [25, 29]. Then, we simulated exponentially growing cells using the three models with noise distributions extracted from our experimental data (Fig. 4a and b, Additional file 14: Figure S9). To make the simulations more comparable to our experiments, we also used our experimentally measured distributions of growth rates and birth sizes. Our measurements of cells under continuous illumination revealed a significant correlation between division and birth volume with a slope of 0.75 (Fig. 5b, *P* < 1 × 10^–7^). Compared to the sizer and timer models, this slope most closely mimicked simulations of the adder model (Fig. 5b, slope *m* = 0.97 ± 0.12) and was inconsistent with simulations of either the sizer or timer models (Fig. 5b). Also consistent with only the adder model, increment volume was uncorrelated with birth volume (Fig. 5c, *m* = –0.25, *P* = 0.07). The timer model predicts in ideal conditions that generation time is independent of birth volume, whereas the adder and sizer models predict similar inverse relationships. The experimentally determined negative slope of –0.79 for generation time with respect to birth volume indicated that smaller cells take longer to divide than larger cells, and was in reasonable agreement with our simulations of the adder model (Fig. 5d, *m* = –0.48 ± 0.05, *P* = 7 × 10^–4^). Finally, normalized differences in generation times between sister cells were negatively correlated to the asymmetry in birth volumes (Additional file 15: Figure S10A, slope = –1.04, *P* = 4 × 10^–8^), indicating that the smaller of the sister cells tended to spend a longer time growing before dividing. The slope was closest to that of simulations based on the adder model (Additional file 15: Figure S10A, slope = –0.72 ± 0.29, *P* = 0.07). Thus, while it remains possible that *Synechocystis* cell-size regulation follows a rule that differs subtly from the adder model, *Synechocystis* growth under continuous illumination is clearly inconsistent with the sizer or timer models.

To determine whether light-dark cycles altered the regulation of cell-cycle timing, we computed generation times ignoring the dark periods (as in Fig. 4c, d, Methods) and performed simulations of each control model, sampling birth volumes and growth rates from our light-dark cycle experiment. As with continuous illumination, slopes of division (Fig. 5e), increment volume (Fig. 5f), and generation time (Fig. 5g) as a function of birth volume were more consistent with the adder model compared to the sizer and timer models. The data for generation time asymmetry and birth volume asymmetry were too noisy to determine the significance of the relationships (Additional file 15: Figure S10B). Thus, *Synechocystis* cell growth and division behaviors under light-dark cycles provide further support against the sizer and timer models, independent of intervening dark intervals.

## Discussion

Cyanobacteria are significantly impacted by light and nutrient status. Hence, studying and modeling their growth kinetics provide a useful paradigm for how complex environmental inputs are integrated into cell-cycle control in photosynthetic microorganisms. To determine growth behaviors, size-control mechanisms, and the role of light in cell-cycle progression, we tracked single-cell growth kinetics of *Synechocystis* in a modified microfluidic cell culture system under continuous illumination and light-dark cycles (Fig. 1). With features such as integrated LED lighting and automated refocusing and image acquisition, our microfluidic cell-culture device allows facile multiplexing and long-term tracking of single cells for days, enabling the study of slow-growing organisms such as *Synechocystis*. Moreover, our device does not constrain the movement or growth directions of cells. This aspect is critical for *Synechocystis* cells, whose division planes rotate by 90° every generation (Fig. 2c), and is in contrast to “mother machine” devices [10] that exploit the one-dimensional elongation of rod-shaped organisms to track cells.

Most previous studies of circadian control in cyanobacteria have used the rod-shaped *Synechococcus elongatus* sp. PCC7942, for which batch cultures were entrained over several light-dark cycles, followed by fluorescence imaging of circadian-clock proteins under continuous illumination [30, 31]. In such experiments, expression levels of circadian genes have been observed to oscillate during intervals classified subjectively as “light” and “dark” [2, 32], suggesting that a direct light input can entrain the system and that expression of circadian genes may gate cell division [31]. However, recent studies have shown that clock genes also respond to the ADP/ATP ratio within the cell, which is a read out of metabolic status determined by rates of photosynthesis during the light period [33]. Thus, cyanobacterial growth and division can also be affected by light through metabolism, and cell behaviors after entrainment but under continuous illumination are likely distinct from phenotypes that emerge after transfer to a dark environment in which energetics also change dramatically. Our microfluidic platform provides the ability to directly observe the growth behavior of single *Synechocystis* cells during the dark phase, with short, low-intensity light exposures. The level of light used is sufficient for accurate cell tracking and demonstrably does not induce any cell growth in the dark (Fig. 3a, b). Furthermore, our custom image analysis pipeline does not require fluorescence labeling of the cell periphery for cell-size quantification, thus reducing stress imposed on cells during imaging. In future experiments, our device would also permit the localization of fluorescently tagged proteins in concert with bright-field imaging.

Under continuous illumination, *Synechocystis* cells followed exponential expansion kinetics at low cell density, which was previously observed in fast-growing coccoid *Staphylococcus aureus* cells [34]. On average, cell size increased slightly over time, which may be due to the transition from batch culture to a surface-associated mode of growth. Twelve-hour dark periods simply suspended growth and division (Fig. 3a, b), but did not alter exponential growth (Additional file 9: Figure S6B), generation times (Fig. 4b), sister-cell generation time correlation (Fig. 4c and d), division symmetry (Fig. 4e, f), or cell-size control (Fig. 5) as compared to cells grown in continuous light. Cells showed no obvious signs at the gross level of growth of anticipating transitions into or out of the dark periods, even after three dark phases. The rapid cessation and resumption of growth when transitioning from light to dark and vice versa, respectively, suggest that light affects biomass accumulation through rapid metabolic control rather than via changes mediated by transcriptional/translational mechanisms, which are typically on the timescale of hours.

Despite substantial variation in growth rates (~30%), sister cell generation times were strikingly similar; for some sisters, the variation in generation times was only a few percent. The positive correlation between sister generation times argues against the uneven partition of molecules (mRNA, proteins, metabolites) as the source of generation time variation because such mechanisms would yield a negative correlation between sister generation times. Sister cells with different generation times tended to result from an asymmetric division (Fig. 4c, e), suggesting that the maintenance of generation times between sisters requires similarity in cellular composition between the two sister cells produced by a symmetric division and that generation times are then determined relatively deterministically (and similarly) in the two sisters. Another study has observed a positive correlation between sister generation times in mammalian cells [35]. One potential explanation for the high degree of correlation between sisters, as compared with that between mother and daughters (Additional file 13: Figure S8C), involves deterministic components shared by sisters that are not inherited. One study argues that an underlying nonlinear process affecting generation time would produce such a correlation, whereby cell divisions occurring during a particular phase of the nonlinear process would produce daughter cells with generation times corresponding to that inherited phase [35]. On the other hand, mother and daughter cells are unlikely to inherit the same phase, and hence would have uncorrelated generation times. In *Synechocystis* cells, a likely candidate for such a nonlinear effect on generation time is the circadian cycle. Although it is possible that phases of the circadian cycle influence cell-cycle duration, resulting in the generation time patterns that we observe, we did not observe any evidence of circadian regulation in our single-cell growth data. Instead, size-based cell-cycle regulation alone tends to produce correlated sister generation times.

By comparing our data with simulations of three size-control models, we determined that compared with the sizer or timer models, *Synechocystis* follows more closely to an adder principle whereby a constant volume is added each cell cycle. This model explains both the strong correlation between generation times of sisters resulting from a symmetric division (Fig. 4c, d), given that their similar size implies that a similar time period is required to accumulate the appropriate volume increment, and the difference in generation times between sisters resulting from an asymmetric division (Fig. 4c, e), with the smaller of the two cells requiring longer to accumulate the volume increment during exponential growth. The adder model also recapitulates many other growth statistics better than the sizer and timer models, including sister asymmetries in both continuous illumination (Additional file 4: Table S1) and during light-dark cycles (Additional file 4: Table S2), although in some cases measurement noise precludes determination of the nature of the correlation and in other cases there were small deviations between the predicted and experimental slopes (Additional file 4: Tables S1 and S2). Molecular mechanisms underlying size regulation via any of the three models have not been determined in any bacterial species. It is possible that certain (perhaps all) species actually implement a combination of cell-size regulation methods, which are in turn controlled by translational and/or metabolic processes. It has been proposed that size control is affected by the dilution of transcription factors or the initiation of DNA replication rather than upon cell division, and that the regulated quantity is cell size per genome or replication origin rather than cell size per se [36, 37]. If this is indeed the case, the fact that *Synechocystis* cells are considered to be polyploid [38] may underlie inconsistencies between our experimental data and simulations based on the adder model.

## Conclusions

Size and growth control are fundamental physiological features of all cells, and tools such as microfluidics and automated image analysis make possible the careful quantification of these parameters with great precision and can be combined with statistical analyses. The ability to image lineages for multiple generations, over several days, potentiates studies in other slow growing cyanobacteria such as *Synechococcus* to address the generality of the behaviors we have uncovered, particularly the immediate responses to changes in light. How cells respond to changes in the environment such as nutrient starvation is not generally understood, and cyanobacteria experience daily light cycles that likely require adaptation of their size and growth; single-cell imaging of such transitions can be a powerful tool to shed light on the underlying control mechanisms [39]. Given the likely commonality of adder-based cell-size control in *Synechocystis* with fast-growing heterotrophs such as *E. coli* and eukaryotes such as *S. cerevisiae*, it is tempting to speculate about the generality of the adder rule in other walled organisms that exhibit size homeostasis such as the shoot apical meristem of plants (or even in wall-less eukaryotes). The diversity of mechanisms for cell-size determination and maintenance from bacteria to eukaryotes suggests that the basis of the adder principle is either a common molecular component across kingdoms, such as DNA, or is based on common physical constraints. Uncovering the molecular mechanisms of size homeostasis, particularly during environmental perturbations, represents an exciting future challenge.

## Methods

### Microfluidic fabrication

The PDMS microfluidic cell culture chip was fabricated using standard multilayer soft lithography techniques [16] (Additional file 16: Figure S11). Briefly, molds were created using SU8 (MicroChem, Massachusetts, USA) or AZ (EMD Performance Materials, Darmstadt, Germany) photoresist. SU8 was used to achieve rectangular-channel cross sections while AZ photoresist was used to create rounded-channel cross-sections that facilitate valve formation. After mold fabrication, PDMS mixture was poured onto the top-layer mold to form a thick block and spun onto the bottom-layer mold to form a thin film. Finally, the two PDMS layers were bonded to each other and to a glass slide. The dimensions of each microfluidic chamber are 1850 × 900 μm, and the height is 25 μm (Additional file 1: Figure S1A). The heights of control and flow channels are 25 and 20 μm, respectively.

### Bulk *Synechocystis* culturing

*Synechocystis* cell culture stocks were grown in glass culture tubes inside a shaking incubator at 30 °C. Tubes were secured at an angle for improved agitation. Cells were grown in BG11 medium (Sigma) under white light (80 μE m^–2^ s^–1^). Cells were not entrained beforehand.

### Field emission scanning electron microscopy (FE-SEM)

*Synechocystis* cells were spotted on polycarbonate membrane filters, after which they were fixed and gently processed using a Vacuum Microfiltration apparatus. Disks were sputter-coated with gold palladium and visualization was performed on a Zeiss Sigma FE-SEM. Images were processed using a noise-reduction algorithm.

### Microfluidic-based *Synechocystis* growth

Before each experiment, the microfluidic chip was flushed with water to passivate uncured PDMS bonds. Flow channels were treated with 2% Pluronic F-127 solution (Sigma-Aldrich, Missouri, USA) for 1 h to prevent cells from sticking to the surfaces during the loading process, which would cause blockage. Pluronic was used to passivate only channel walls and was rinsed away prior to cell loading; the microfluidic chambers used for culturing cells were not treated with Pluronic solution. Chambers were treated with 0.01% polylysine (PLL; Sigma-Aldrich, Missouri, USA) for 2 h to promote attachment of cells to PDMS surfaces and to facilitate imaging [40]. PLL was only used to treat PDMS culturing chambers and was washed away after 2 h of incubation. Due to the push-up valve structure of the microfluidic device, culturing chambers have PDMS surfaces on all sides, which likely also results in less PLL adsorption compared with a glass surface.

Exponentially growing cells cultured in a flask under continuous illumination were seeded into the chambers at low cell density with each field of view initially containing approximately 50–100 cells, so that cells would experience an approximately constant environment for a long period of time; indeed, we observed that growth rate remained constant for the first ~30 h (Fig. 1c). Even though the capacity of each chamber is approximately 40 nL, only the center portion (400 × 400 μm) was imaged at the higher magnification (20×) used to image bacterial cells. Cultures were not entrained before introduction into the microfluidic device. During all experiments, 5% CO_2_ was continuously added into the microscope’s environmental chamber through a water bubbler, which helped to maintain humidity and CO_2_ concentration. For light-dark cycle experiments, LEDs were switched on and off every 12 h. Toward the end of the experiments, cell growth, as measured by combining all lineages, slowed down (Fig. 1c), but growth was not significantly affected within the cell cycle of individual cells (Fig. 2e). The slowdown in growth is likely due to nutrient depletion; we did not continuously flush the channels with new medium to avoid washing out cells from the chambers, which would have made it challenging to track cells for several generations. Interestingly, we found that sister cell generation times were highly correlated in both the first and last 30 h of the experiment (Additional file 13: Figure S8A, B), indicating that the mechanism driving this correlation was unaffected by the slowdown in growth.

### Image acquisition and analysis

Bright-field images were acquired every 10 min from 20 chambers of the microfluidic chip with 20× magnification (Leica DMI 6000, Illinois, USA). The image exposure time (16 ms) did not affect cell growth, as demonstrated by the lack of growth during the dark phase of light-dark experiments (Fig. 3a, b). A custom MATLAB-based script coordinated all components of the cell culture system including the microscope, stage, camera, and LEDs. Every hour, contrast-based autofocusing was performed to correct for vertical stage drift. Automated image analysis was performed with custom MATLAB scripts. Details of acquisition and analysis methods can be found in the Additional file 4 [41].

### Cell volume calculation

To reconstruct the volume of the cell from cell contours extracted from time-lapse images, we assumed rotational symmetry with respect to the major axis of the cell or doublet (Additional file 5: Figure S4). For each cell, we first extracted the orientation of the major axis. Total cell volume was then calculated by summing the volumes of a series of circular disks with radii equal to the perpendicular distances between the major axis and the cell boundary.

### Extraction of growth parameters from single-cell growth curves

For single-cell growth curves obtained from experiments under continuous illumination, we extract birth size, division size, and generation time following Additional file 12: Figure S7. In order to analyze single-cell growth curves obtained from experiments under light-dark cycles, we removed segments corresponding to dark periods of the experiment. The fact that neither growth nor division is observed in dark allowed us to piece together single-cell growth curve segments from only the illuminated periods and still obtain continuous growth curves. In this case, generation time corresponds to the total amount of time a cell spends under light.

To determine how much of the observed correlation between sister cell generation times (Fig. 4c, d) could be attributed to their time of birth, we bootstrapped the generation-time differences, randomizing generation times based on cells having either similar birth times or similar division times (as determined by binning with a 5-h bin width). The standard deviation of unrandomized sister cell generation time differences was 3% of their mean generation time, whereas the standard deviation was 8.3% for randomly selected cell pairs in a similar growth window. The distributions after randomizing differed significantly from our data, indicating that sister cell generation times are highly correlated.

### Simulations of cell division models

Distributions of birth volumes, cell division asymmetry, and growth rates were used as inputs for simulations of the sizer, adder, and timer models of cell size regulation. We observed an increase in cell size (Fig. 2e) that occurred largely in the first full cell cycle; cell birth and cell division sizes were approximately consistent for later cell cycles (Additional file 14: Figure S9). We then directly incorporated measurements of the size increase into our simulations using the model described in Additional file 4. Details of our simulation methodology are included in Additional file 4.

## Additional files

Additional file 1: Figure S1. Microfluidic system setup. (a) Schematic of the microfluidic cell culture chip. This device is a two-layer push-up device. Blue lines show outline of flow channels containing cell culture chambers and reagent inlets. Yellow lines outline the control layer containing push-up valves, multiplexer, and a peristaltic pump. Right: image of an actual cell culture chip. The dimensions of each chamber are 1.85 × 900 mm, and the height is 25 μm. Due to the higher magnification (20×) used to image bacteria, only the center portion (0.3 × 0.3 mm) of the chip is monitored. (b) Illustration of control and operation of the microfluidic cell culture chip. The device is surrounded by an environmental chamber and secured on an automated stage of a Leica DMI 6000 microscope. Each control channel is connected with an independent pressure reservoir located above the microscope. All associated equipment, including CO_2_ and temperature control, is connected to a computer and the operation of each component is controlled automatically via custom MATLAB software. (c) The cell culture chip contains 16 inputs that are routed to a central pump and distributed to 96 chambers. Each chamber is independently addressable. (PDF 6 MB)
Additional file 2: Figure S2. Data analysis pipeline of growth videos. Features from training images are used to extract clusters from each frame. Clusters in adjacent images are then associated with each other. During this process, we account for the possibility of cell growth and division. Temporal association is used to construct cell lineages from which growth statistics are generated. (PDF 77 kb)
Additional file 3: Figure S3. Probabilistic image analysis pipeline. (a–d) Outline features extracted from the training dataset. These distributions are used to generate probability distributions for classifying single cells, doublets, and clusters of more than two cells. (a) Distributions of cluster areas in the training set corresponding to single cells (blue), doublets (red), or more than two cells (green). (b) Distributions of cluster circularity (Eq. 1 in Additional file 4). A perfect circle and a line have circularities of 1 and 0, respectively. (c) Distributions of cluster eccentricity, defined as the ratio of the distance between the two foci of an ellipse to the major axis. A perfect circle and a line have eccentricities of 0 and 1, respectively. (d) Distribution of distance between cell centers for doublets. (e) Cluster correlations between adjacent images in time. Distance metric (Eq. 3 in Additional file 4) guarantees that closer clusters are more correlated with each other. White circles show features such as movement and division between adjacent temporal frames. (PDF 499 kb)
Additional file 4: Additional Methods and **Tables S1**–**S3.** **Table S1.** Summary of simulations of cells grown under continuous illumination. **Table S2.** Summary of simulations of cells grown under 12-h light-dark illumination cycles. **Table S3.** Parameters used for cell-size regulation simulations. (DOCX 56 kb)
Additional file 5: Figure S4. Method of cell volume extraction from bright-field images. Since the image of each cell represents its two-dimensional projection, we assume that each cell is rotationally symmetric with respect to its major axis. Based on this assumption, to compute the volume of each cell (or doublet), we first extract the orientation of its major axis. Then, we add up the volumes of circular disks perpendicular to the major axis with thicknesses of one pixel. (PDF 354 kb)
Additional file 6: Figure S5.
*Synechocystis* cells are ellipsoidal and most lineages grow in microfluidic chambers. (a) Two images representing the same field of view of a microfluidic chamber at an early time point (green) and 40 h later (maroon). Growth is evident for most (if not all) *Synechocystis* lineages. (b) Bright-field image of *Synechocystis* cells imaged on a glass slide, illustrating their ellipsoidal shape. (c) Quantification of the ellipticity of cells and cell doublets in (b), defined as the ratio of minor axis length to major axis length, demonstrates that most cells are not spherical. (PDF 2 MB)
Additional file 9: Figure S6. Growth behavior is similar across all chambers in light-dark cycle experiment. (a) Total growth in different chambers under light-dark cycles. Substantial growth is observed during illuminated periods across all microfluidic chambers. In the dark, minimal growth is detected. (b) Residual errors (gray, with mean shown in black) of exponential fits to lineage growth curves during the illumination periods L1 and L2 of Fig. 3a. (PDF 366 kb)
Additional file 12: Figure S7. Representative *Synechocystis* single-cell growth curves. (a) A representative single-cell growth curve under continuous illumination, showing birth volume (*V*_*b*_), division volume (*V*_*d*_), and generation time (*T*). (b) A representative single-cell growth curve during light-dark growth. To compute generation time, the 12-h dark periods were removed. (PDF 347 kb)
Additional file 13: Figure S8. Robust sister-sister generation time correlation under continuous illumination. (a, b) The generation times of sister cells were highly correlated for cells that divided in the first 30 h of the experiment (a) and in the last 30 h (b). The similarity between the two correlation coefficients demonstrates that the correlation between sister-cell generation times is not an artifact of temporal variations in cell growth. (c) There was no significant correlation between the generation times of mother and daughter cells. (PDF 340 kb)
Additional file 14: Figure S9. Cells were slightly smaller when grown under light-dark cycles. (a, b) Left: birth (a) and division (b) volumes as a function of time for cells grown under continuous illumination (blue) and light-dark cycles (orange). Right: volume distributions. There is no birth-volume data in the dark since cell division did not occur in the dark (Fig. 3d). (PDF 400 kb)
Additional file 15: Figure 10. Sister cell asymmetry data from simulations of cell growth using the sizer (orange), timer (purple), and adder (yellow) models. Slopes of relationships between generation time asymmetry and birth volume asymmetry were compared with experimental data (gray circles) and their least square linear fit (black). Birth volumes were normalized by mean birth volume and generation times were normalized by mean generation time. (a) Under continuous illumination, generation time asymmetry and birth volume asymmetry between sister cells were negatively correlated with a slope of –1.04. This slope matched most closely to simulations performed with the adder model (slope = –0.72 ± 0.29 SD), indicating that the sister cell of a pair with the smaller birth volume tended to spend a longer time growing before dividing. (b) Experimental data for growth under light-dark cycles was too noisy to reveal a significant relationship. (PDF 339 kb)
Additional file 16: Figure S11. Microfluidic cell culture chip fabrication process. To make the cell culture chip, photoresist was used to build structures on silicon wafers, using standard photolithography to create molds. PDMS was poured over the flow-layer mold and spun on the control-layer mold. Both molds were then bonded together on a glass slide. (PDF 286 kb)
