# Supplementary material for: Long-term microfluidic tracking of coccoid cyanobacterial cells reveals robust control of division timing
Source: BMC Biol. 2017 Feb 14;15:11. doi: 10.1186/s12915-016-0344-4 (PMC5310064; doi:10.1186/s12915-016-0344-4)
Supplement: Additional file 1: Figure S1. — Microfluidic system setup. (a) Schematic of the microfluidic cell culture chip. This device is a two-layer push-up device. Blue lines show outline of flow channels containing cell culture chambers and reagent inlets. Yellow lines outline the control layer containing push-up valves, multiplexer, and a peristaltic pump. Right: image of an actual cell culture chip. The dimensions of each chamber are 1.85 × 900 mm, and the height is 25 μm. Due to the higher magnification (20×) used to image bacteria, only the center portion (0.3 × 0.3 mm) of the chip is monitored. (b) Illustration of control and operation of the microfluidic cell culture chip. The device is surrounded by an environmental chamber and secured on an automated stage of a Leica DMI 6000 microscope. Each control channel is connected with an independent pressure reservoir located above the microscope. All associated equipment, including CO2 and temperature control, is connected to a computer and the operation of each component is controlled automatically via custom MATLAB software. (c) The cell culture chip contains 16 inputs that are routed to a central pump and distributed to 96 chambers. Each chamber is independently addressable. (PDF 6 MB) [file 12915_2016_344_MOESM1_ESM.pdf]

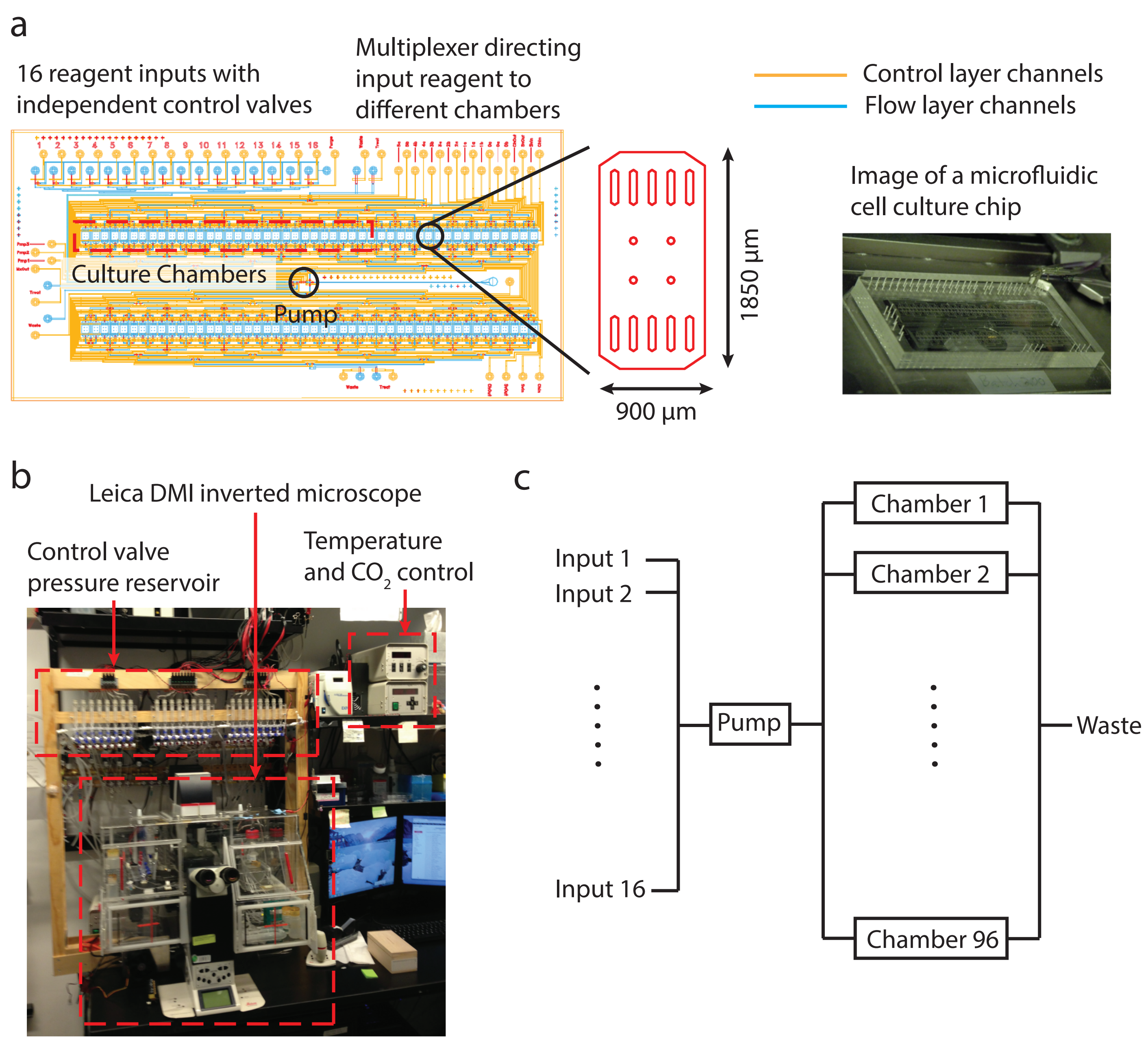
a
16 reagent inputs with independent control valves
Multiplexer directing input reagent to different chambers
Control layer channels
Flow layer channels
Culture Chambers
Pump
1850 µm
900 µm
Image of a microfluidic cell culture chip
b
Leica DMI inverted microscope
Control valve pressure reservoir
Temperature and CO_2 control
c
Input 1
Input 2
Input 16
Pump
Chamber 1
Chamber 2
Chamber 96
Waste
