# Supplementary material for: Long-term microfluidic tracking of coccoid cyanobacterial cells reveals robust control of division timing
Source: BMC Biol. 2017 Feb 14;15:11. doi: 10.1186/s12915-016-0344-4 (PMC5310064; doi:10.1186/s12915-016-0344-4)
Supplement: Additional file 2: Figure S2. — Data analysis pipeline of growth videos. Features from training images are used to extract clusters from each frame. Clusters in adjacent images are then associated with each other. During this process, we account for the possibility of cell growth and division. Temporal association is used to construct cell lineages from which growth statistics are generated. (PDF 77 kb) [file 12915_2016_344_MOESM2_ESM.pdf]

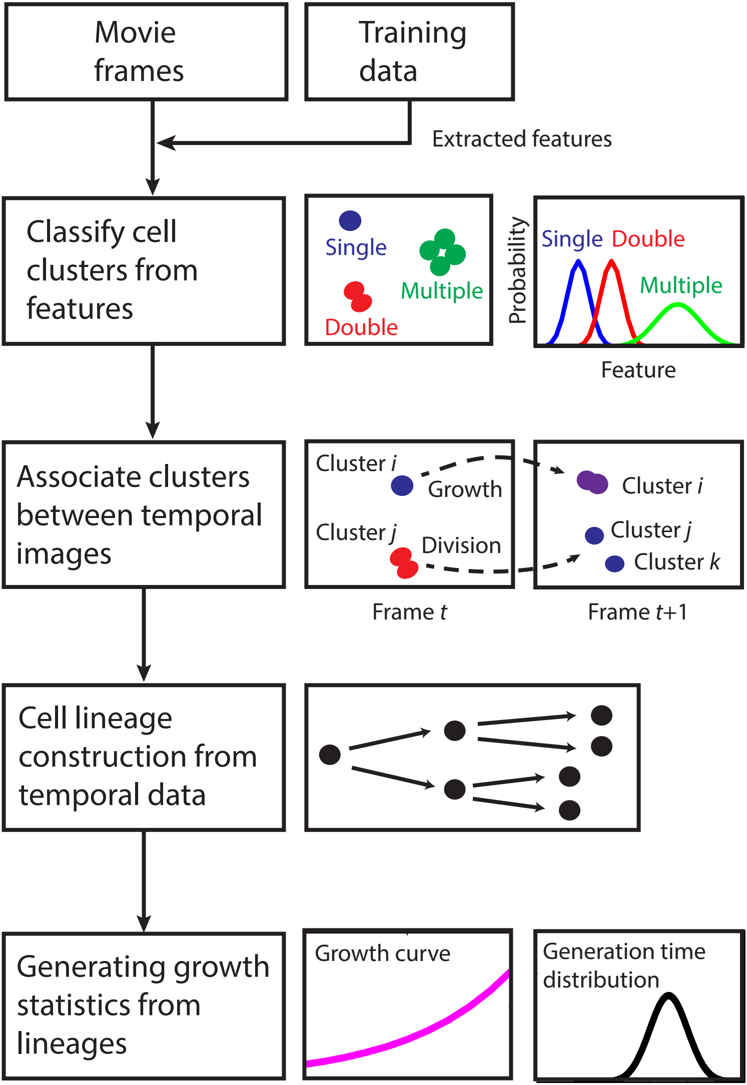
Movie frames
Training data
Extracted features
Classify cell clusters from features
Single
Multiple
Double
Probability
Single
Double
Multiple
Feature
Associate clusters between temporal images
Cluster i
Growth
Cluster j
Division
Cluster i
Cluster j
Cluster k
Frame t
Frame t+1
Cell lineage construction from temporal data
Generating growth statistics from lineages
Growth curve
Generation time distribution
