# Supplementary figures and images for: Long-term microfluidic tracking of coccoid cyanobacterial cells reveals robust control of division timing
Source: BMC Biol. 2017 Feb 14;15:11. doi: 10.1186/s12915-016-0344-4 (PMC5310064; doi:10.1186/s12915-016-0344-4)

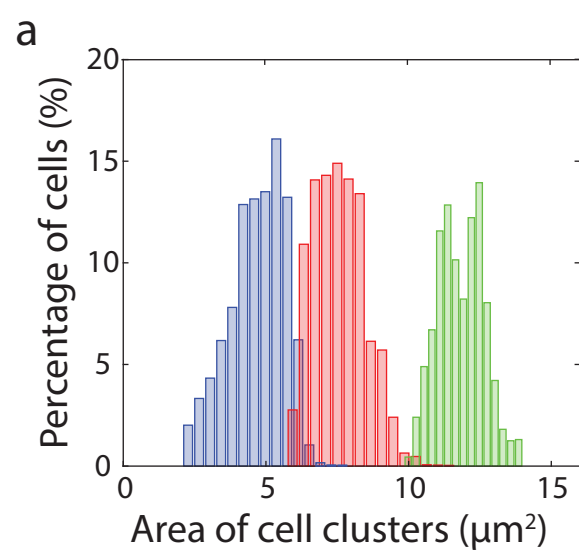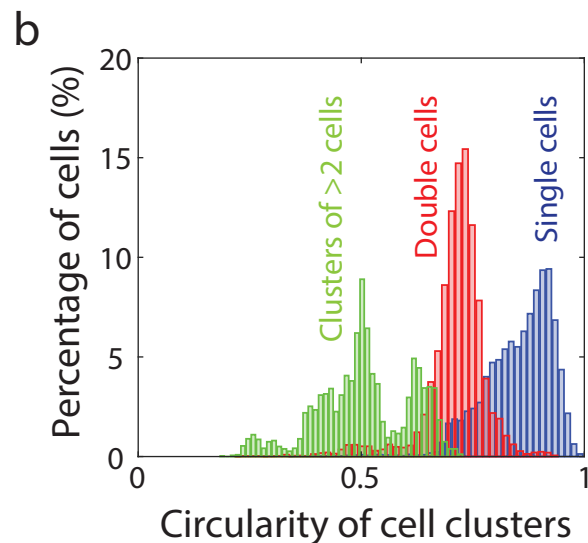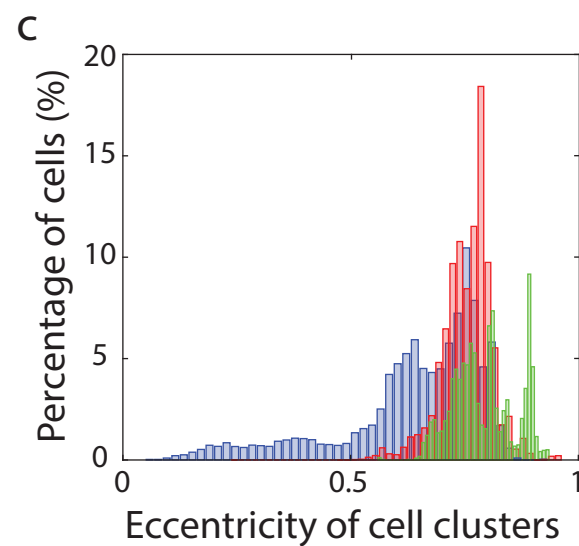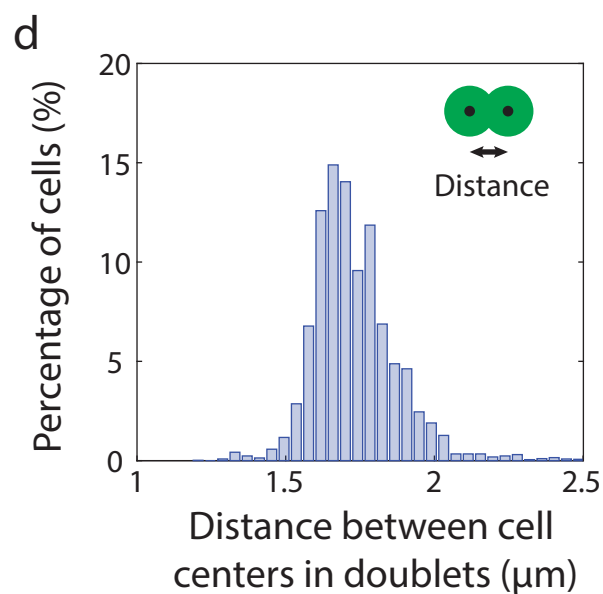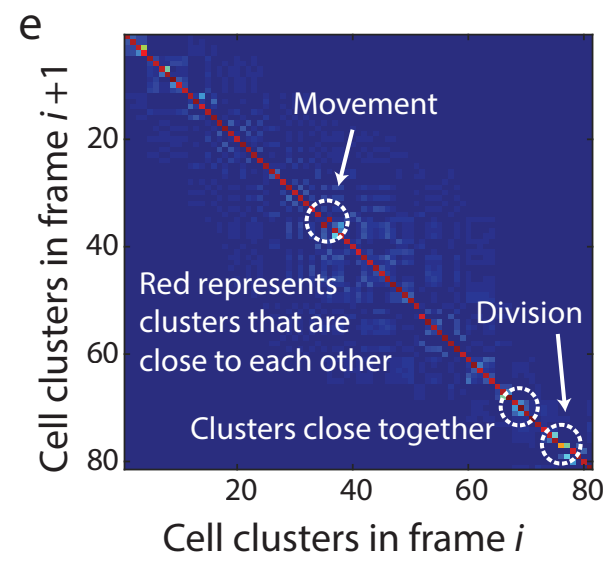

Supplement: Additional file 3: Figure S3. — Probabilistic image analysis pipeline. (a–d) Outline features extracted from the training dataset. These distributions are used to generate probability distributions for classifying single cells, doublets, and clusters of more than two cells. (a) Distributions of cluster areas in the training set corresponding to single cells (blue), doublets (red), or more than two cells (green). (b) Distributions of cluster circularity (Eq. 1 in Additional file 4). A perfect circle and a line have circularities of 1 and 0, respectively. (c) Distributions of cluster eccentricity, defined as the ratio of the distance between the two foci of an ellipse to the major axis. A perfect circle and a line have eccentricities of 0 and 1, respectively. (d) Distribution of distance between cell centers for doublets. (e) Cluster correlations between adjacent images in time. Distance metric (Eq. 3 in Additional file 4) guarantees that closer clusters are more correlated with each other. White circles show features such as movement and division between adjacent temporal frames. (PDF 499 kb) [file 12915_2016_344_MOESM3_ESM.pdf]

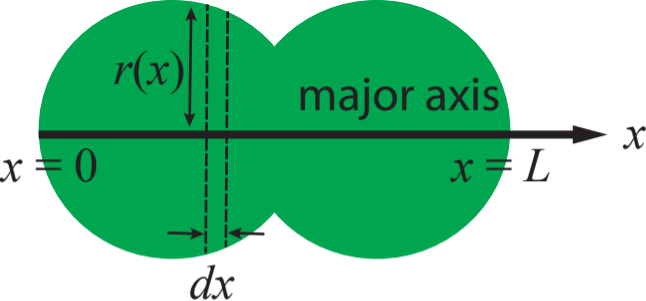

$$V = \int_{x=0}^L \pi r(x)^2 dx$$

Supplement: Additional file 5: Figure S4. — Method of cell volume extraction from bright-field images. Since the image of each cell represents its two-dimensional projection, we assume that each cell is rotationally symmetric with respect to its major axis. Based on this assumption, to compute the volume of each cell (or doublet), we first extract the orientation of its major axis. Then, we add up the volumes of circular disks perpendicular to the major axis with thicknesses of one pixel. (PDF 354 kb) [file 12915_2016_344_MOESM5_ESM.pdf]

a

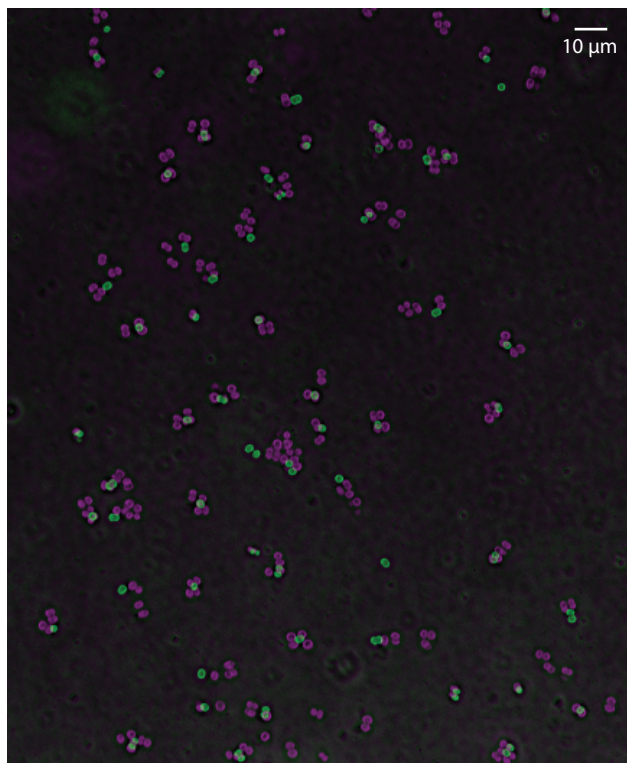

b

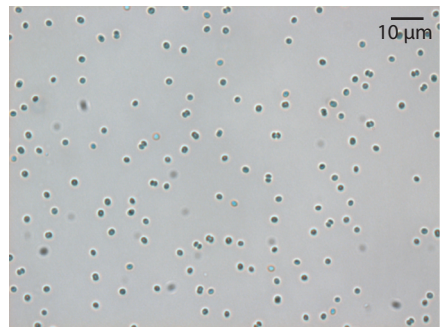

c

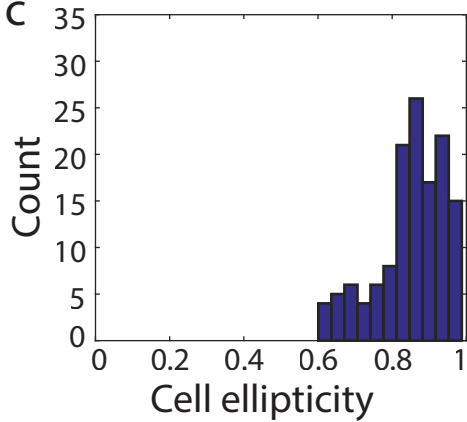

Supplement: Additional file 6: Figure S5. — Synechocystis cells are ellipsoidal and most lineages grow in microfluidic chambers. (a) Two images representing the same field of view of a microfluidic chamber at an early time point (green) and 40 h later (maroon). Growth is evident for most (if not all) Synechocystis lineages. (b) Bright-field image of Synechocystis cells imaged on a glass slide, illustrating their ellipsoidal shape. (c) Quantification of the ellipticity of cells and cell doublets in (b), defined as the ratio of minor axis length to major axis length, demonstrates that most cells are not spherical. (PDF 2 MB) [file 12915_2016_344_MOESM6_ESM.pdf]

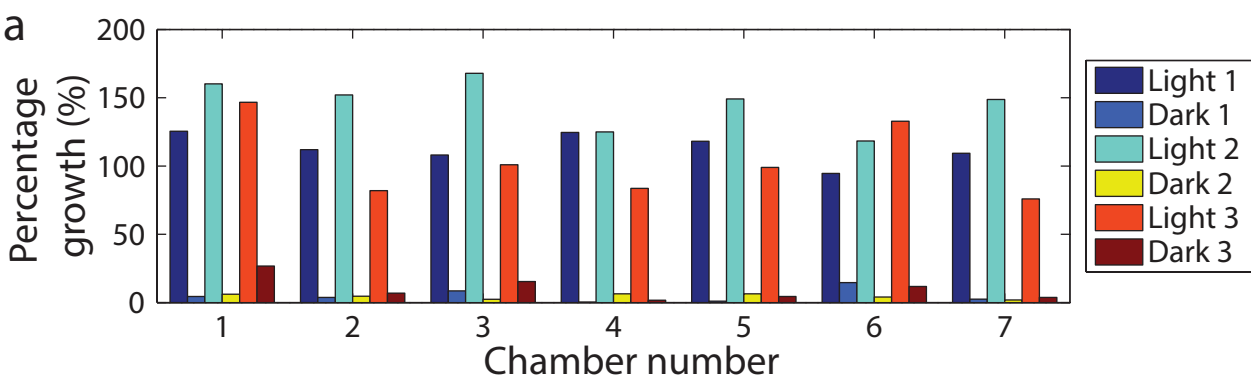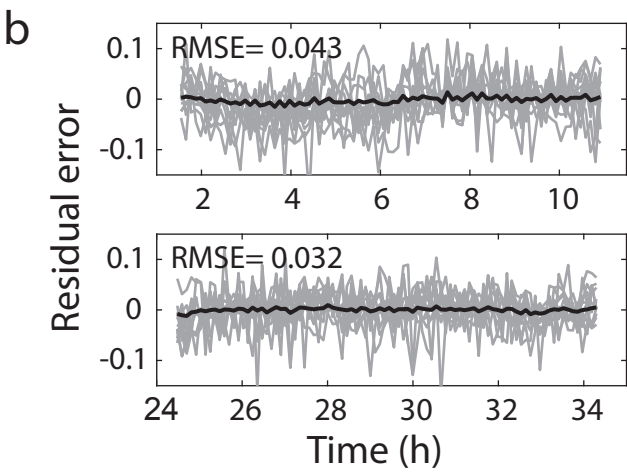

Supplement: Additional file 9: Figure S6. — Growth behavior is similar across all chambers in light-dark cycle experiment. (a) Total growth in different chambers under light-dark cycles. Substantial growth is observed during illuminated periods across all microfluidic chambers. In the dark, minimal growth is detected. (b) Residual errors (gray, with mean shown in black) of exponential fits to lineage growth curves during the illumination periods L1 and L2 of Fig. 3a. (PDF 366 kb) [file 12915_2016_344_MOESM9_ESM.pdf]

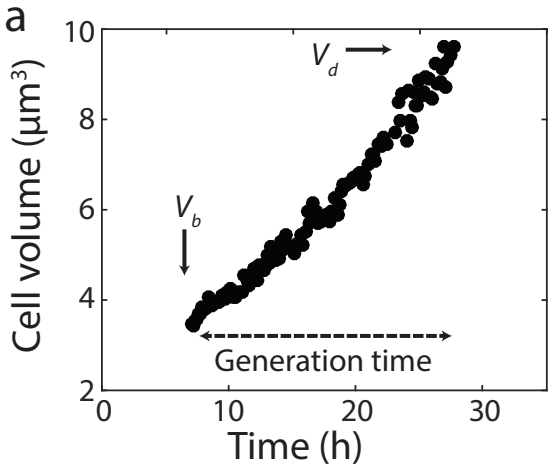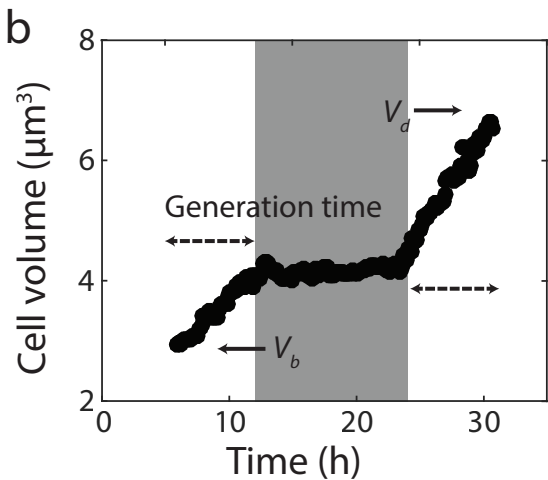

Supplement: Additional file 12: Figure S7. — Representative Synechocystis single-cell growth curves. (a) A representative single-cell growth curve under continuous illumination, showing birth volume (V b), division volume (V d), and generation time (T). (b) A representative single-cell growth curve during light-dark growth. To compute generation time, the 12-h dark periods were removed. (PDF 347 kb) [file 12915_2016_344_MOESM12_ESM.pdf]

a

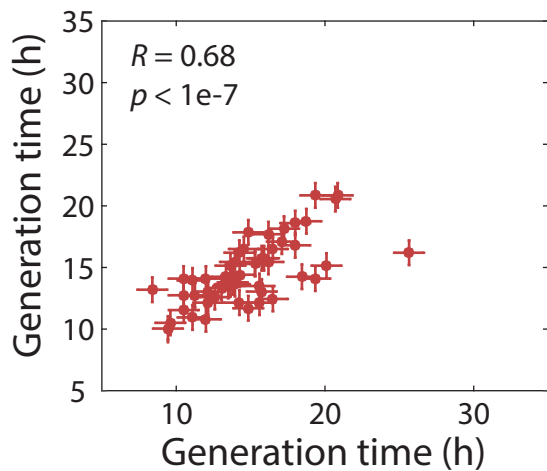

b

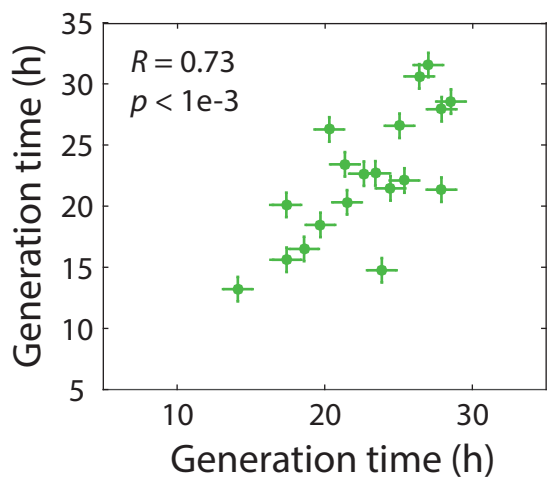

c

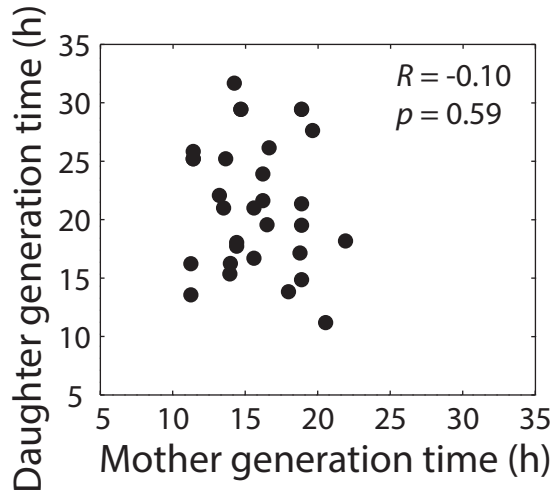

Supplement: Additional file 13: Figure S8. — Robust sister-sister generation time correlation under continuous illumination. (a, b) The generation times of sister cells were highly correlated for cells that divided in the first 30 h of the experiment (a) and in the last 30 h (b). The similarity between the two correlation coefficients demonstrates that the correlation between sister-cell generation times is not an artifact of temporal variations in cell growth. (c) There was no significant correlation between the generation times of mother and daughter cells. (PDF 340 kb) [file 12915_2016_344_MOESM13_ESM.pdf]

a

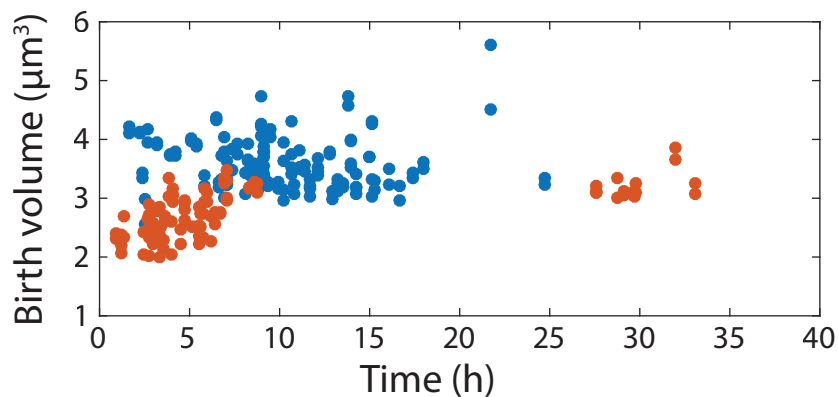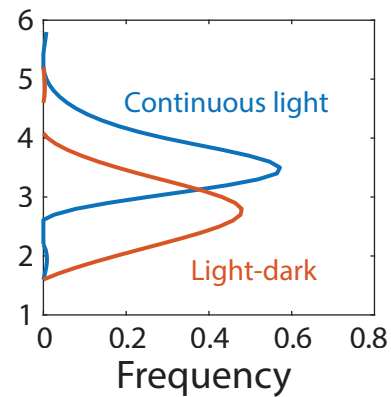

b

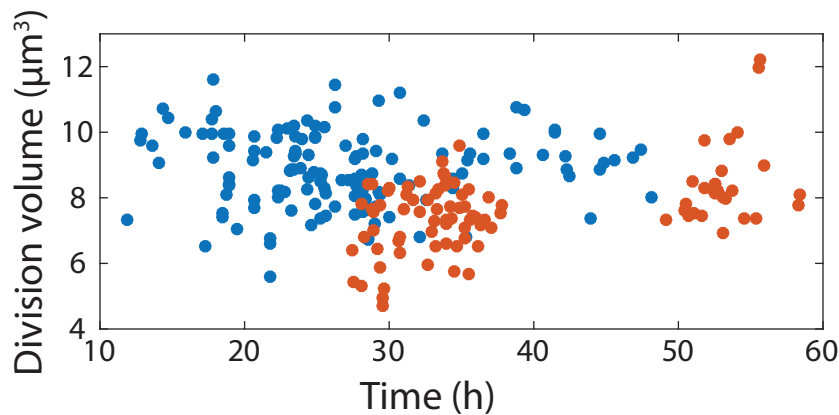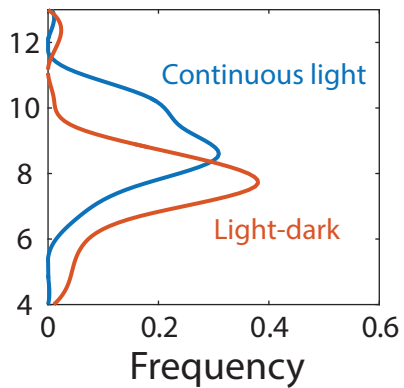

Supplement: Additional file 14: Figure S9. — Cells were slightly smaller when grown under light-dark cycles. (a, b) Left: birth (a) and division (b) volumes as a function of time for cells grown under continuous illumination (blue) and light-dark cycles (orange). Right: volume distributions. There is no birth-volume data in the dark since cell division did not occur in the dark (Fig. 3d). (PDF 400 kb) [file 12915_2016_344_MOESM14_ESM.pdf]

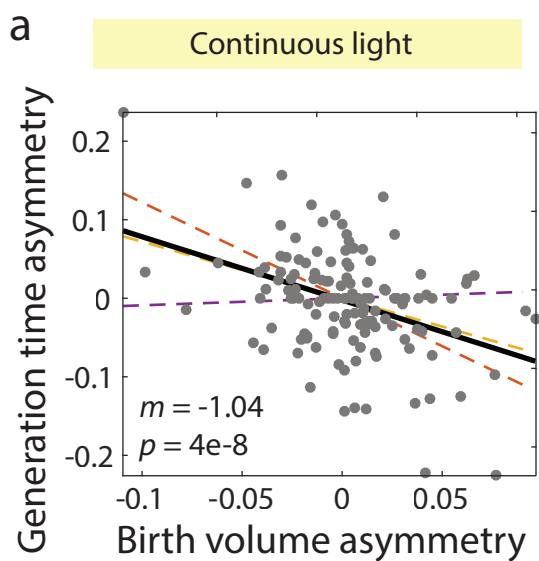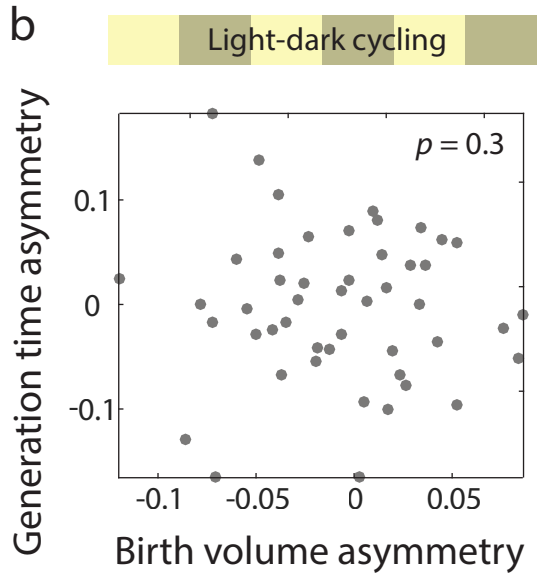

Supplement: Additional file 15: Figure 10. — Sister cell asymmetry data from simulations of cell growth using the sizer (orange), timer (purple), and adder (yellow) models. Slopes of relationships between generation time asymmetry and birth volume asymmetry were compared with experimental data (gray circles) and their least square linear fit (black). Birth volumes were normalized by mean birth volume and generation times were normalized by mean generation time. (a) Under continuous illumination, generation time asymmetry and birth volume asymmetry between sister cells were negatively correlated with a slope of –1.04. This slope matched most closely to simulations performed with the adder model (slope = –0.72 ± 0.29 SD), indicating that the sister cell of a pair with the smaller birth volume tended to spend a longer time growing before dividing. (b) Experimental data for growth under light-dark cycles was too noisy to reveal a significant relationship. (PDF 339 kb) [file 12915_2016_344_MOESM15_ESM.pdf]

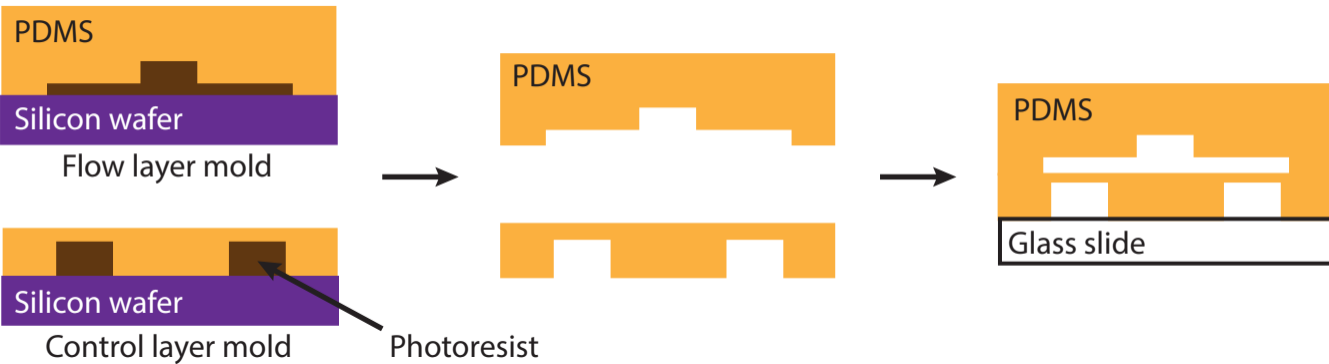

Supplement: Additional file 16: Figure S11. — Microfluidic cell culture chip fabrication process. To make the cell culture chip, photoresist was used to build structures on silicon wafers, using standard photolithography to create molds. PDMS was poured over the flow-layer mold and spun on the control-layer mold. Both molds were then bonded together on a glass slide. (PDF 286 kb) [file 12915_2016_344_MOESM16_ESM.pdf]
