# Supplementary material for: Long-term microfluidic tracking of coccoid cyanobacterial cells reveals robust control of division timing
Source: BMC Biol. 2017 Feb 14;15:11. doi: 10.1186/s12915-016-0344-4 (PMC5310064; doi:10.1186/s12915-016-0344-4)
Supplement: Additional file 4: — Additional Methods and Tables S1–S3. Table S1. Summary of simulations of cells grown under continuous illumination. Table S2. Summary of simulations of cells grown under 12-h light-dark illumination cycles. Table S3. Parameters used for cell-size regulation simulations. (DOCX 56 kb) [file 12915_2016_344_MOESM4_ESM.docx]

**Supplementary Information for “Long-term microfluidic tracking of coccoid cyanobacterial cells reveals robust control of division timing”**

Feiqiao Brian Yu^a,b^, Lisa Willis^b,c^, Rosanna Chau^b^, Alessandro Zambon^b,d^, Mark Horowitz^a^, Devaki Bhaya^e,1^, Kerwyn Casey Huang^b,f,1^, Stephen Quake^b,g,1^

^a^Department of Electrical Engineering, Stanford University, Stanford, CA 94305, USA

^b^Department of Bioengineering, Stanford University, Stanford, CA 94305, USA

^c^Sainsbury Laboratory, Cambridge University, Cambridge, England CB2 1LR

^d^Department of Industrial Engineering, University of Padova, Padova 35131, Italy

^e^Department of Plant Biology, Carnegie Institution for Science, Stanford, CA 94305, USA­­

^f^Department of Microbiology and Immunology, Stanford University School of Medicine, Stanford, CA 94305, USA

^g^Howard Hughes Medical Institute, Chevy Chase, MD 20815, USA

*^1^Correspondence*: [dbhaya@stanford.edu](mailto:dbhaya@stanford.edu), [kchuang@stanford.edu](mailto:kchuang@stanford.edu), [quake@stanford.edu](mailto:quake@stanford.edu)

**Supplementary Methods**

**Microfluidic cell culture system for cyanobacteria**

To monitor growth of *Synechocystis* cells, we modified a microfluidic-based cell culture setup (16) that provides a stable growth environment with the option to perform high-throughput culturing and automated imaging. The system consists of components that control and image a two-layer PDMS microfluidic chip (Fig. S11). A Leica DMI inverted microscope and a Qimaging camera are used for time-lapse imaging. The microfluidic chip is maintained inside an environmental chamber with constant temperature and humidity. Fluidic switches and tubing are used to transfer pressure and control on-chip push-up valves. The cell culture chip measures 5 cm long and 2 cm wide, and contains 96 chambers in which independent experiments can be performed (Fig. S1A). These 96 chambers are independently addressed via a fluidic multiplexer consisting of 24 valves (Fig. S1A-C). There are 16 input channels to which culturing media and buffer reagents can be connected (Fig. S1A,C) for multiplexing.

The system was modified to accommodate *Synechocystis* growth requirements that include providing light for photosynthesis and extended incubations times since these cells have division times on the scale of hours. Leveraging the transparency of PDMS, white LEDs (light emitting diode) were added above the microfluidic chip to provide a light source for the cyanobacteria (Fig. 1A). In addition, polylysine coating was used inside the PDMS chambers to facilitate cell adhesion (40). A custom MATLAB script controls all components. One technical advantage of our image acquisition script is the ability to search for the focal plane periodically to offset stage drift. This improvement enables monitoring of all culture experiments without human intervention for days to weeks.

**Image analysis pipeline**

The image analysis pipeline is divided into two branches. The training branch takes as input a small subset of frames from a time-lapse video (Fig. S2). Each image in the set of video frames was subjected to a maximally stable extremal regions (MSER) segmentation algorithm, which is insensitive to small background variations and deviations from the focal plane (41); as a result, artifacts such as non-uniform lighting and small variations in the distance from the objective to the imaging place from frame to frame do not significantly affect the quality of processed data. Morphological operations were then performed to remove small artifacts from the black and white image. Result of the segmentation step is a black and white image in which white pixels represent *Synechocystis* cells whereas black regions represent the background. Following segmentation, regions of connected white pixels were grouped into clusters. Because *Synechocystis* cells often grow in close proximity to each other, each cluster extracted from segmented images may represent more than one cell. Based on the set of training images, each cluster was classified as containing one, two, or more than two cells. Distributions of features of clusters, including area (Fig. S3B), circularity (Eq. 1; Fig. S3C), and eccentricity (Fig. S3D), belonging to each group were extracted:

$\text{Circularity}=\frac{4\pi Area}{\mathrm{Perimeter}^{2}}$. (1)

For clusters containing two cells, we computed the distribution of distances between the cell centers (Fig. S3E). The mean and variance of all feature distributions from the training dataset were used in probability assignments during the main branch of the image analysis pipeline.

In the main branch of our image analysis pipeline, videos were first subjected to individual frame analysis. After MSER segmentation, each cluster of white pixels was assigned three probabilities corresponding to the cluster containing one, two, or more than two cells. Each probability was calculated as a product of feature probabilities using the distributions obtained from the training data (Eq. 2). Rather than making a definitive classification for each cluster at the present stage in the pipeline, the assignment of probabilities could be modified by later considerations described below when classification decisions are ambiguous.

$P_{\mathrm{cluster}}=P_{\mathrm{area}}P_{\mathrm{eccentricity}}P_{\mathrm{circularity}}$ (2)

After individual frame analysis, the analysis pipeline assigns temporal cluster associations. To correctly utilize temporal data, clusters were linked across successive frames using a distance metric (Eq. 3). When cluster centers from adjacent frames are close, the distance metric is ~1. When cluster centers are far away, the distance metric is close to zero. Thus, this distance metric can be viewed as a probability that cell clusters from adjacent images represent the same cluster at different times. A cluster from one frame may be closest to multiple clusters in the following frame. In such cases, an area constraint was enforced that requires areas of linked regions in adjacent images be similar. An example of such situation is when two cells move apart. The region containing both cells in the previous frame is now associated with two regions in the following frame, each including one cell.

$P_{\mathrm{dist}}=\exp\left( -\left\| \mathrm{Center}_{\mathrm{frame}i}-\mathrm{Center}_{\mathrm{frame}i+1} \right\| \right)$ (3)

Finally, cell numbers are assigned on the set of associated clusters. Using typical distance distributions statistics between cell centers of doublets (Fig. S3E), the most probable number of cells in a cluster was computed for each frame. Again, due to noise, this number could vary from frame to frame. By integrating the probabilities of cluster identity and cell number from multiple successive frames and requiring that cell numbers only increase at a slow rate, the likelihood of assigning the wrong number of cells to any cluster was significantly reduced. After cell number determination, the cells within the cluster were defined by a modified version of the k-means clustering algorithm. Cell centroids were then computed based on the k-means results.

**Simulations of cell-cycle statistics for different modes of division regulation**

We extended a phenomenological model (26, 29) that generates cell-size and generation time distributions and correlations according to the sizer, adder, or timer mode of cell-size regulation to assess which mode best recapitulates our data. For a given cell volume at birth $V_{b}$ and cell growth rate $\alpha$, the simulation generates a corresponding cell size at division $V_{d}$ and a generation time $T$ from the stochastic equations

$$\frac{V_{d}}{\mu_{V_{b}}}=f\frac{V_{b}}{\mu_{V_{b}}}+\left( 2-f+2\Delta\right)+\epsilon$$

$\mathrm{where} \left\{ \begin{aligned} f=0 \mathrm{gives} \mathbf{sizer} \mathrm{rule} \\ f=1 \mathrm{gives} \mathbf{adder} \mathrm{rule} \end{aligned} \right.\mathrm{or} \frac{V_{d}}{\mu_{V_{b}}}=\frac{V_{b}}{\mu_{V_{b}}}e^{\alpha\mu_{T}}+\epsilon\mathrm{gives}\mathbf{timer} \mathrm{rule}$,

and $\frac{T}{\mu_{T}}=\frac{1}{\alpha\mu_{T}}\log\left( \frac{V_{d}}{V_{b}} \right)$

where $\mu_{V_{b}}$ is the mean cell birth volume; $\Delta$ is the mean fractional change between birth volumes of successive generations (in our data, which includes only the first pair of complete cell cycles from each lineage acquired over the course of the experiment, there is a mean increase in volume of 25% over the cell cycle, giving $\Delta$ = 0.25); $\epsilon$ is a normally distributed random variable with mean 0 and standard deviation *σ* where

$\sigma=\sqrt{\mathrm{Var}\left( \frac{V_{d}}{\mu_{V_{b}}} \right)-f^{2} \mathrm{Var}\left( \frac{V_{b}}{\mu_{V_{b}}} \right)}$,

for sizer and adder models. For the timer model, $\sigma$ was chosen such that standard deviation of the simulated cell division volume agrees with experimental values, and $\mu_{T}$ is the mean generation time. Finally, *α* is the growth rate. There is no time dependence of birth volume or division volume (Fig. S9A,B) indicating that $\Delta$ is not a function of time. The parameters $\Delta$, *σ*, $\mu_{V_{b}}$, and $\mu_{T}$ were extracted directly from experimental data and were fixed for all simulations (Table S3).

­

For a given mode of division regulation and either the continuous light or light-dark cycle data, a division volume and a generation time were simulated for each experimentally determined cell birth volume and corresponding cell growth rate. Pearson correlation coefficients and slopes among these variables were then computed along with *p*-values derived from Student’s t-test representing the confidence of the linear fit slopes for the simulated or experimental output (Table S1 and Table S2). This procedure was repeated 100 times to compute the mean and standard deviation of each statistic. In addition, median *p*-values were also computed for all simulations. Using these parameters, we assessed whether the given mode of division (sizer, adder, timer) recapitulates our experimental data.

Table S1: Summary of simulations of cells grown under continuous illumination.

*m* indicates the slope of the relationship. Standard deviation is shown for all simulated slopes and correlation coefficients.

|  |  |  | Experimental data | Sizer  model | Adder model | Timer  model |
| --- | --- | --- | --- | --- | --- | --- |
| 1 | Sister generation time correlation | *p* | 2.00e-40 | 3.00e-15 | 5.00e-15 | 3.00e-01 |
|  |  | *R* | 0.86 | 0.62 ± 0.06 | 0.62 ± 0.07 | 0.02 ± 0.12 |
| 2 | Generation time vs. birth-volume asymmetry | *p* | 4.00e-08 | 8.00e-03 | 7.00e-02 | 4.00e-01 |
|  |  | *m* | -1.04 | -1.22 ± 0.35 | -0.72 ± 0.29 | 0.09 ± 0.40 |
| 3 | Division volume vs. birth volume | *p* | 1.00e-07 | 5.00e-01 | 1.00e-12 | 2.00e-09 |
|  |  | *m* | 0.75 | 0.03 ± 0.13 | 0.97 ± 0.12 | 2.14 ± 0.10 |
| 4 | Increment volume vs. birth volume | *p* | 7.00e-02 | 2.00e-10 | 5.00e-01 | 1.00e-03 |
|  |  | *m* | -0.25 | -0.97 ± 0.13 | -0.03 ± 0.12 | 1.14 ± 0.10 |
| 5 | Generation time vs. birth volume | *p* | 5.00e-12 | 8.00e-10 | 7.00e-04 | 5.00e-01 |
|  |  | *m* | -0.79 | -0.92 ± 0.07 | -0.48 ± 0.05 | 0.02 ± 0.06 |
| 6 | Increment volume vs. division volume | *p* | 7.00e-100 | 3.00e-108 | 3.00e-98 | 5.00e-207 |
|  |  | *m* | 0.86 | 1.00 ± 0.02 | 0.82 ± 0.02 | 0.94 ± 0.00 |
| 7 | Generation time vs. division volume | *p* | 6.00e-01 | 2.00e-12 | 2.00e-06 | 6.00e-07 |
|  |  | *m* | 0.03 | 0.44 ± 0.06 | 0.28 ± 0.05 | 0.05 ± 0.01 |
| 8 | Generation time vs. increment volume | *p* | 4.00e-04 | 3.00e-20 | 3.00e-12 | 3.00e-07 |
|  |  | *m* | 0.19 | 0.52 ± 0.05 | 0.45 ± 0.06 | 0.05 ± 0.01 |

Table S2: Summary of simulations of cells grown under 12-h light-dark illumination cycles.

*m* indicates the slope of the relationship. Standard deviation is shown for all simulated slopes and correlation coefficients.

|  |  |  | Experimental data | Sizer  model | Adder model | Timer  model |
| --- | --- | --- | --- | --- | --- | --- |
| 1 | Sister generation time correlation | *p* | 2.00e-17 | 2.00e-04 | 1.00e-03 | 4.00e-01 |
|  |  | *R* | 0.9 | 0.53±0.15 | 0.46±0.17 | 0.00±0.20 |
| 2 | Generation time vs. birth-volume asymmetry | *p* | 3.00e-01 | 1.00e-01 | 2.00e-01 | 4.00e-01 |
|  |  | *m* | -0.22 | -0.90±0.42 | -0.64±0.38 | 0.21±0.78 |
| 3 | Division volume vs. birth volume | *p* | 9.00e-04 | 4.00e-01 | 2.00e-03 | 4.00e-13 |
|  |  | *m* | 1.07 | 0.02±0.32 | 1.02±0.28 | 4.55±0.23 |
| 4 | Increment volume vs. birth volume | *p* | 8.00e-01 | 3.00e-03 | 6.00e-01 | 2.00e-09 |
|  |  | *m* | 0.07 | -0.98±0.32 | 0.02±0.28 | 3.55±0.23 |
| 5 | Generation time vs. birth volume | *p* | 3.00e-08 | 1.00e-09 | 7.00e-07 | 4.00e-01 |
|  |  | *m* | -1.06 | -1.81±0.14 | -1.40±0.12 | 0.04±0.14 |
| 6 | Increment volume vs. division volume | *p* | 5.00e-48 | 4.00e-49 | 5.00e-48 | 8.00e-87 |
|  |  | *m* | 0.89 | 1.00±0.03 | 0.90±0.03 | 0.90±0.01 |
| 7 | Generation time vs. division volume | *p* | 7.00e-03 | 4.00e-05 | 1.00e-02 | 1.00e-03 |
|  |  | *m* | 0.17 | 0.41±0.09 | 0.23±0.09 | 0.06±0.02 |
| 8 | Generation time vs. increment volume | *p* | 2.00e-06 | 2.00e-09 | 1.00e-05 | 4.00e-04 |
|  |  | *m* | 0.31 | 0.54±0.07 | 0.41±0.09 | 0.07±0.02 |

Table S3: Parameters used for cell-size regulation simulations.

|  | Continuous-light simulations | Light-dark cycle simulations |
| --- | --- | --- |
| Birth-volume asymmetry noise | 0.016 | 0.023 |
| Target-size shift | 0.239 | 0.373 |
| Sizer model division-volume noise | 0.329 | 0.484 |
| Adder model division-volume noise | 0.306 | 0.458 |
| Timer model division-volume noise | 0.222 | 0.356 |
